# Estimation of sodium and potassium intakes assessed by two 24-hour urine collections in a city of Indonesia

**DOI:** 10.1017/S0007114521000271

**Published:** 2021-11-28

**Authors:** Dianis Wulan Sari, Maiko Noguchi-Watanabe, Satoshi Sasaki, Junaiti Sahar, Noriko Yamamoto-Mitani

**Affiliations:** 1 Department of Gerontological Home Care and Long-Term Care Nursing, Division of Health Sciences and Nursing, Graduate School of Medicine, The University of Tokyo, 7-3-1 Hongo, Bunkyo-ku, Tokyo 113-0033, Japan; 2 Department of Community and Gerontological Nursing, Faculty of Nursing, Universitas Airlangga. Jln. Mulyorejo, East Java 60115, Surabaya, Indonesia; 3 Department of Social and Preventive Epidemiology, School of Public Health, The University of Tokyo, 7-3-1 Hongo, Bunkyo-ku, Tokyo 113-0033, Japan; 4 Faculty of Nursing, Universitas Indonesia, Jln. Prof. Dr. Bahder Djohan, West Java 16424, Depok, Indonesia

**Keywords:** 24-Hour urine, Intake, Sodium, Potassium, Urine excretion

## Abstract

Intakes of excess Na and insufficient K are two major contributors of heart diseases and stroke development. However, no precise study has previously been carried out on Na and K intakes among Indonesian adults. The present study aimed to estimate the Na and K intakes using two consecutive 24-h urine collections. Participants were community-dwelling adults aged between 20 and 96 years, randomly selected from a pool of resident registration numbers. Of the 506 participants, 479 (240 men and 239 women) completed urine collections. The mean Na excretion was 102·8 and 100·6 mmol/d, while the mean K excretion was 25·0 and 23·4 mmol/d for men and women, respectively. Na and K excretions were higher in participants with a higher BMI. A higher K excretion was associated only with younger age. More than 80 % of the participants consumed more than 5 g/d of salt (the upper limit recommended by the Indonesian government), whereas none of them consumed more than 3510 mg/d of K (the lower limit). The high Na and low K intakes, especially high Na among participants with high BMI, should be considered when future intervention programmes are planned in this country.

In Indonesia, the common non-communicable diseases that lead to death are stroke, heart disease and cancer^(1)^. Excess Na intake and insufficient K intake have been generally reported as factors associated with the development of stroke and heart disease^(2–5)^. However, consensus has not been reached regarding the association between Na intake, blood pressure and heart disease^(5–8)^. The findings of previous studies have not been consistent with regard to the association between high and low Na intake and mortality related to heart disease^(7–13)^. Although the definitions of ‘high’ and ‘low’ for Na intake are unclear^(8)^, the average daily consumption of Na is between 3·0 and 6·0 g/d globally^(14–16)^, and many countries and regions have instituted salt reduction programmes.

Before intervention programmes for reducing Na and increasing K intakes are discussed, considered or planned, actual intakes of which reliability is high enough should be referred. Nationwide studies including those conducted in the UK, Japan, Spain, Italy, Turkey and Slovenia^(17–22)^ have reported estimations of Na and K intakes based on 24-h urine to evaluate the progress of salt reduction programmes^(17–25)^. However, thus far, there has been no nationwide survey of Na and K intakes in the Indonesian population. Hitherto, only two studies have evaluated Na and K intakes among Indonesians, both with methodological limitations. One study used 24-h dietary recall^(26)^, and the other study used a 24-h urinary analysis in a small and single-sex sample^(27,28)^.

Several methods have been used to evaluate Na and K intakes, including food consumption questionnaires^(29)^, spot urine collection^(30)^ and 24-h urine collection^(18,20,31,32)^. Because 86 % of Na and 77 % of K consumed through the diet are excreted in the urine over 24 h^(33)^, the 24-h urine collection is considered the ‘gold standard’ method for Na and K intake measurements, in individuals and populations^(34)^. Since it is necessary to evaluate Na and K intakes in a representative sample of individuals, a community-based approach may be appropriate for Indonesian people, especially those living in sub-urban and rural areas^(35)^.

The present study aimed to precisely determine the Na and K intakes of people living in a city of Indonesia by evaluating in two consecutive 24-h urine collections.

## Methods

### Study design and study area

This cross-sectional descriptive correlational study was conducted in a city of East Java, Indonesia. The prevalence of hypertension, stroke and heart disease in this province was higher than the national prevalence of these diseases^(35,36)^. The present study was conducted in the city of Nganjuk, as it is representative of a sub-urban area and is a flat, non-coastal city in Indonesia. Data collection for this survey was carried out by home visits. To increase the reliability and the convenience in collecting data to facilitate the analyses of the urine, only one city was selected for the study.

### Study participants and sampling methods

The participants were community-dwelling young (aged 20–59 years) and older adults (aged ≥ 60 years). The age group cut-offs were based on the standard values set by the Indonesian government^(37)^. The exclusion criteria were as follows: unable to provide informed consent, difficulties with memory or communication, following a diet prescribed by a doctor or a dietitian at the time of the study or within 1 year prior to the study, fasting, severe diseases (i.e. kidney failure, total paralysis and liver disease) and presence of other conditions could cause difficulties in the 24-h urine collection. We obtained the residents’ data from the city office. In randomising, the groups were classified by age group and sex.

The main investigator used a computer-based random number generator, to select the sample from a pool of the residents’ identity card numbers. We calculated the sample size based on the study population for Na levels^(38)^. A previous study recommended a minimum sample size of 120 individuals per sex stratum to detect an approximately 1 g reduction in salt intake over time, based on 24-h urinary Na excretion, with a sd of 75 mmol/d (α = 0·05, power = 0·80)^(38)^. To compensate for possible attrition (e.g. nonparticipation or implausible values), which may have accounted for as high as 10 % of participants, up to 132 people per group stratum (in total 528) were invited to participate.

The present study was conducted according to the guidelines laid down in the Declaration of Helsinki and all procedures involving human subjects/patients were approved by the ethical committee of The University of Tokyo (approval number, 12015). Written informed consent was obtained from all participants. All the participants whose data were collected received an incentive at the end of the data collection.

### Data collection

**Fig. 1.**
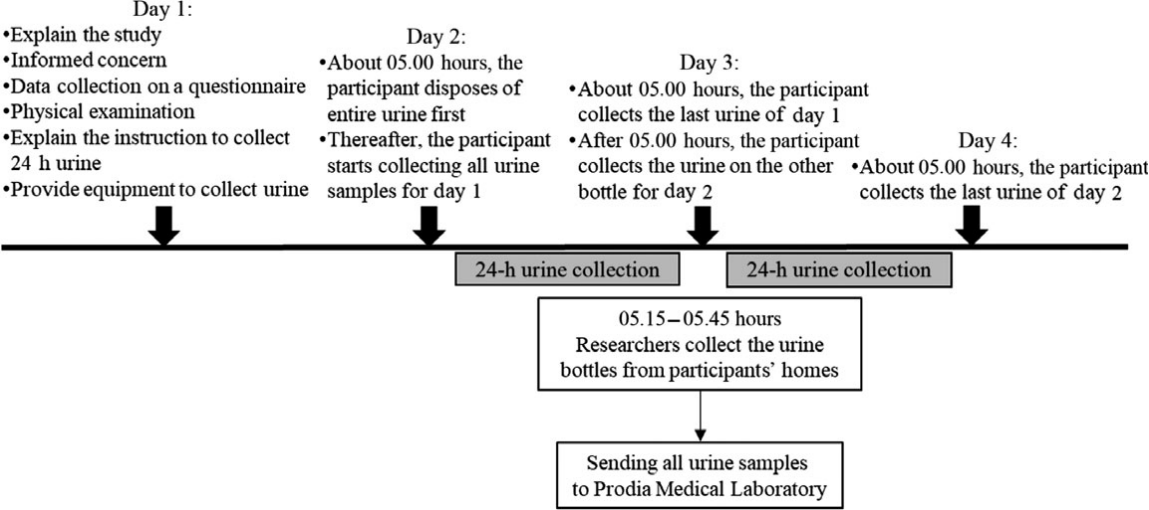
Process of data collection.

All data were collected between November 2018 and September 2019. The data collection process required 4 d per participant (Fig. 1). On the first day, the investigator visited the resident’s home and provided the information about the study. We confirmed the inclusion and exclusion criteria of the participants while providing an explanation of the study. People who agreed to participate provided written consent. Next, the data regarding the participant’s characteristics was collected, and a physical examination was conducted. We then provided instructions on the 24-h urine collection process and on how to complete the self-recorded 24-h urine collection sheet. All equipment was provided by the researcher. On the second to fourth days, the participants began the urine collection at their home. The adverse event in urine collection and other measurement was confirmed on the fourth day.

#### Participants’ characteristics

The section in the questionnaire on participants’ characteristics was developed using the STEPS instrument^(39)^ and used to obtain data on age, sex, education level, medical history, occupation, current drug consumption, tobacco use and alcohol consumption. In the older adult population, activities of daily living were measured using the Barthel Index^(40)^. The Mini-Cog^©(41)^, which is used mainly in the older population, was used to measure the cognitive status. We measured activity patterns using the physical activity levels (PAL) from the long version of the International Physical Activity Questionnaire^(42)^. We estimated the PAL value by summing the result of the time spent on each of a range of activities with various exercise intensities and metabolic equivalent of task values for each activity^(43)^. The International Physical Activity Questionnaire has been validated for use in many countries and translated into many languages^(44)^.

#### 24-h Urine collection

We estimated daily Na and K intakes based on the 24-h urinary excretion. The 24-h urine collection was performed on two consecutive days, using a protocol based on the WHO/Pan American Health Organization report and based on a previous study conducted in Japan^(18,38)^. The urine collection start time (around 05.00 hours) was adjusted to the Indonesian culture for the morning prayer. For urine collection day 1, the participants discarded the first morning urine at around 05.00 hours and, thereafter, all the urine samples were collected. Day 1 collection was completed the next day at the same time (05.00 hours). For day 2, at 05.00 hours, the participants collected the last specimen of day 1; therefore, they did not need to discard the urine to start day 2. The participants needed to change only the bottle for day 2 collection after 05.00 hours. However, we made it flexible for participants to start urine collection between 04.45 and 05.30 hours. If the participant changed the start time, then they would finish at the corresponding end time. We distributed four 1-litre bottles/d. The participants were asked to carry a plastic bottle when they went out and to keep the bottle away from sunlight and cold places. They had separate bottles ‘for home use’ and ‘for outside the home’. Disposable plastic and paper cups were used for easy urine collection, before the urine was poured into the bottle.

We collected the urine from the participants’ homes at approximately 05.15–05.30 hours to ensure the acquisition of urine samples. We asked the participants to repeat the urine collection process when the following conditions occurred: (1) the duration of urine collection exceeded 24 h and 30 min, and (2) participants reported that they had forgotten to collect the urine more than once or were ‘missing more than a few drops and could not estimate the amount’. A previous study that used participants’ self-reporting of missed urine collection mentioned that this method is reliable for measuring completeness of 24-h urine in large population-based biomarker studies^(45)^. Based on previous population surveys, it is preferable to ask participants to repeat the collection if it is potentially incomplete, to ensure complete and accurately timed 24-h urine collection. Such methods were used in the INTERSALT and INTERMAP studies^(46,47)^.

We provided a self-reporting sheet, for the participants to record the start and finish times, as well as the estimated frequency and volume of urine that they forgot or failed to collect. We included the estimated amount along with the actual volume of urine collected. The overall total amount of urine was calculated by Prodia Medical Laboratory, after adjusting for collection times based on the collection records. This method was validated using the para-aminobenzoic acid check method^(48)^.

The Prodia Medical Laboratory used the ISE indirect Na-K-Cl for Gen.2; the ISE module of the Roche/Hitachi cobas c system (Roche Diagnostic) for the quantitative determination of Na and K in urine using ion-selective electrodes. CREP2 creatinine plus ver.2 was used (*in vitro* test (enzyme test)) for the quantitative determination of creatinine concentration in urine on the cobas c 111 system (Roche Diagnostic). Na and K excretions and the Na:K ratio were calculated as follows:

Total 24-h urine excretion (mmol/d) = adjusted volume of 24-h urine (ml/d) × concentration (mmol/ml).

Note: 1 mmol of Na is equal to approximately 58·5 mg of NaCl (salt)

1 mmol of K is equal to approximately 39·1 mg of K

Na:K ratio = 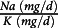


#### Physical examination and environment

Blood pressure was measured using the clinical blood pressure technique^(49,50)^. The measurement was conducted three times using electronic monitors (Omron HEM-7130 with cuff from Omron HEM-CR24 by Omron Healthcare Co. Ltd.) as validated by Takahashi *et al.* ^(51)^. The protocol for blood pressure measurement was based on the British Hypertension Society guidelines and the WHO/Pan American Health Organization. Participants rested for 1 min between each of the readings^(38,52,53)^. The heart rate was also measured three times. The body weight and height were used to calculate BMI. Body weight (kg) was measured using a portable digital scale (Omron HN-286 by Omron Healthcare Co. Ltd.) in units of 100 g and capacity 180 kg. Body height (cm) was measured using a portable stadiometer to the nearest 0·1 cm and capacity 2 m. The weight scale was calibrated daily before each use.

Temperature and humidity have an effect on 24-h urine excretion. However, it was difficult to measure the climate for the 24 h of the individual collections. Therefore, we decided to collect data on temperature and humidity using a thermometer and a hygrometer (Citizen THD501; Citizen System Co. Ltd.), respectively, once daily at 10.00 hours in randomly selected homes in the community area. Almost all the homes of the participants did not have air conditioning.

All the measurements were made according to a standardised protocol by well-trained researchers.

### Statistical analysis

The completeness of the 24-h urine collection was confirmed before the analysis. First, we examined the completeness of the urine collection using two equations. Day 1 (*n* 484) used Joossens *et al.*’s equations^(48,54)^. Joossens *et al.* used a value of <0·6 (urinary creatinine (mmol/d) × 113)/(21 × body weight (kg)). If the ratio of urinary creatinine (mg/d) to body weight (kg) calculated for collection was <60 % or >140 % of the expected value, then that urine sample was excluded from the analysis. Therefore, ninety-nine urine samples (i.e. <60 %, 84 samples and >140 %, 15 samples) were excluded from the day 1 collection. The creatinine value was not available for the day 2 urine collection. Therefore, for the day 2 collection (*n* 482), we used the equations based on the urine volume and self-reported urine collection. Samples with <250 ml total volume, long (>26-h) or short (<22-h) urine collection period or unknown amount of urine loss were excluded. The combination of urine volume and the self-reported method has been verified by para-aminobenzoic acid recovery in an earlier study^(55)^. We excluded seven samples from day 2.

Two types of urinalyses were performed. The first was categorised by sex using the data collected from as many participants as possible (successful samples from day 1 or 2, or both, collections). When both samples were considered complete, the mean value was used; when only one collection was considered complete, its value was considered the representative one. This decision was made based on the results of previous studies and using concordance analyses (Bland–Altman, linear regression, Cohen’s κ and Spearman analyses)^(18,56)^. The first analysis included 479 participants (240 men and 239 women). In the second type of urinalysis, the distribution of the habitual Na and K excretions was calculated only for participants with two successful 24-h urine collections. The second analysis included 380 participants (204 men and 176 women).

We analysed the data for men and women separately due to the different urine excretions between sexes caused by factors such as dietary pattern, age, BMI and PAL^(18,19,57)^. The difference in the participants’ characteristics was analysed using the Student’s *t* test and *χ* ^2^ test. We then calculated the Cr ratio, as of the observed to the expected Cr excretion, as described by Joossens *et al*.^(58)^. Na and K excretions were then calculated as the excretion of the electrolytes per 1 g excretion of creatinine. Additionally, the Na and K excretions ratio was calculated and expressed as mg/d.

A multivariable linear regression model was used to examine the association between age, BMI, and PAL and urine excretion. To compare the effect of each covariate quantitatively, we used standardised partial regression coefficients. The distribution of habitual intake of Na and K in the analysed population was simulated using HabitDist software version 1.2 based on the best-power method^(59,60)^ to account for day-to-day variation^(61)^. This programme estimated the means and standard deviations and distribution (10th, 25th, 50th, 75th and 90th percentiles) of the usual Na and K excretions (as well as the proportion above or below the defined cut-off values). For the cut-off values, we used the WHO and Indonesian government recommendations for decreasing salt and improving K intake^(62–64)^. Based on the estimation reported in a previous study that 86 % of Na and 77 % of K consumed orally ware excreted into the urine^(33)^, the intake recommendation was adjusted by multiplication with 0·86 for Na and 0·77 for K^(59,61)^.

Additionally, we analysed the association between the environment and urine excretion using the Student’s *t* test. All analyses were performed using SPSS version 26 (IBM). *P* < 0·05 was considered to be statistically significant.

## Results

**Fig. 2.**
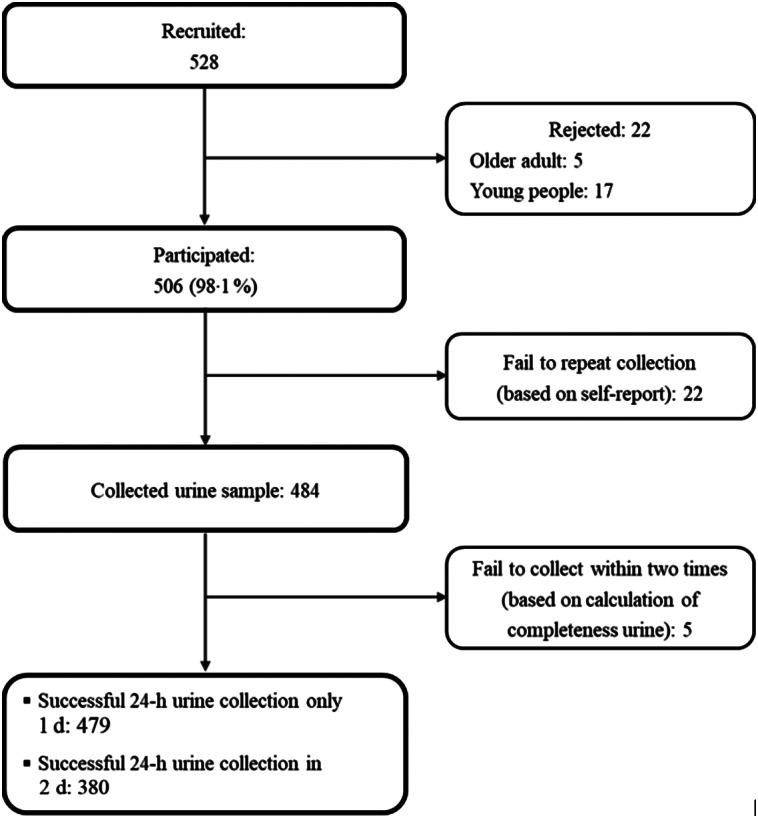
Flow chart of the study participants.

We recruited 528 people, and twenty-two people refused to participate in the present study; finally, 506 individuals completed the questionnaire. Of these, 484 took part in the 24-h urine collection (Fig. 2). Participants’ characteristics are summarised in Table 1. We included 240 men and 239 women with a mean age of 56·5 years. Among all participants, 36·7 % were diagnosed with a non-communicable disease, with the prevalence of hypertension in men and women being 24·6 and 34·3 %, respectively. In addition, the mean systolic blood pressure and diastolic blood pressure were 139 and 87·4 mmHg, respectively. Moreover, the mean BMI was 22·3 kg/m^2^, with significant differences observed between men and women participants.

**Table 1.** Characteristics of the study participants (Mean values and standard deviations; numbers and percentages)

	Total (*n* 479)		Men (*n* 240)		Women (*n* 239)		
	Mean/*n*	sd/%	Mean/*n*	sd/%	Mean/*n*	sd/%	*P* *
Age (years)	56·5	14·7	57·0	14·8	56·0	14·6	0·49
Completed secondary school (yes)	345	72·0	186	77·5	159	66·5	0·007
Married	374	78·1	204	85·0	170	71·1	<0·001
Occupation (current)							<0·001
Employee	48	10·0	32	13·3	16	6·7	
Trade	81	16·9	55	22·9	26	10·9	
Farmer	152	31·7	120	50·0	32	13·4	
Housewife	139	29·0	0	0	139	58·2	
Others	17	3·5	12	5·0	5	2·1	
Unemployed (unable to work)	42	8·8	21	8·8	21	8·8	
Diagnosed have NCD (yes)†	176	36·7	79	32·9	97	40·6	0·08
Having multiple NCD (yes)†	14	2·5	7	2·9	7	2·9	0·99
The primary NCD†							
Do not know/do not have a disease	303	63·3	161	67·1	142	59·4	0·10
Hypertension	141	29·4	59	24·6	82	34·3	0·03
Diabetes	7	1·5	3	1·3	4	1·7	0·50
Cardiovascular	5	1·0	4	1·7	1	0·4	0·37
Cancer	1	0·2	0	0·0	1	0·4	1·00
Urinary tract stone	1	0·2	1	0·4	0	0·0	1·00
Chronic gastritis	1	0·2	0	0·0	1	0·4	1·00
Hyperuricaemia	1	0·2	1	0·4	0	0·0	1·00
Hyperthyroid	3	0·6	2	0·8	1	0·4	1·00
Others	16	3·3	9	3·8	7	2·9	0·40
Medication within 5 d before urine collection (yes)	24	5·0	14	5·8	10	4·2	0·54
Not consuming drug	455	95·0	226	94·2	229	95·8	
Antihypertensive drugs	18	3·8	13	5·4	5	2·1	
Diuretics	1	0·2	0	0·0	1	0·4	
Antihyperglycaemic agent	1	0·2	0	0·0	1	0·4	
Antiplatelet drug	1	0·2	0	0·0	1	0·4	
Antianginal drug	1	0·2	1	0·4	0	0·0	
Supplement/herbal	1	0·2	0	0·0	1	0·4	
Nootropic drug	1	0·2	0	0·0	1	0·4	
Smoking habit and consuming alcohol							
Smoker (yes)	144	30·1	144	60·0	0	0·0	<0·001
Number of cigarettes (*n* 140)							
1–6 cigarettes/d			33	23·6			
7–12 cigarettes/d			92	65·7			
> 13 cigarettes/d			15	10·7			
Past smoker (yes)	179	37·4	179	74·6	0	0·0	<0·001
Couple is smoker (yes)	140	29·2	1	0·4	139	58·2	<0·001
Consuming alcohol (yes)	2	0·4	2	0·8	0	0·0	<0·001
Blood pressure (mmHg)							
SBP||	139·3	24·7	139·9	23·9	138·7	25·5	0·60
DBP||	87·4	11·2	87·4	11·8	87·3	10·7	0·87
HR (times/min)‡	82·1	9·6	81·2	9·2	83·0	9·8	0·04
Ht (cm)	155·5	7·3	159·8	5·9	151·1	6·0	<0·001
Wt (kg)	53·8	10·5	53·5	10·4	54·2	10·7	0·46
BMI (kg/m^2^)	22·3	4·2	20·9	3·7	23·7	4·2	<0·001
Underweight (<18·5)	93	19·4	64	26·7	29	12·1	<0·001
Normal (18·5–24·9)	270	56·4	146	60·8	124	51·9	
Overweight (25·0–29·9)	99	20·7	27	11·3	72	30·1	
Obesity I (30·0–34·9)	17	3·5	3	1·3	14	5·9	
Barthel index score, independent (*n* 240)	236	98·3	117	97·5	119	99·2	0·44
PAL (MET × h)	71·2	56·7	67·2	56·4	75·2	56·7	0·12
Environment§							
Temperature (°C)	27·5	0·4	27·6	0·5	27·5	0·5	0·02
Humidity (%)	56·9	3·4	56·5	3·5	57·2	3·2	0·02

NCD, non-communicable disease; SBP, systolic blood pressure; BBP, diastolic blood pressure; HR, heart rate; Ht, height; Wt, weight; PAL, physical activity level; MET, metabolic equivalents.

* The statistic was based on Student’s *t* test and *χ* ^2^ test.

† This NCD was reported by participant and only diagnosed cases; therefore, undiagnosed not included.

‡ It was measured three times; this value was mean of three times measurements.

§ It was measured once per d at 10.00 hours; this value was the mean of two measurements.

|| It was measured three times; this value was the mean of the second and third measurements.

Table 2 showed the result of concordance analysis for day 1 and 2 urine excretions. The mean difference among the 2 d collection of Na and K was 3·05 (95 % CI –0·96, 7·07) and 0·78 (95 % CI 0·05, 1·5), respectively. Despite this, the limits of agreement of Na and K were −75 to 81 and −13·3 to 14·9, respectively. From the Spearman analysis, the *r* (*P* value) of Na and K for the 2 d collection was 0·63 (<0·001) and 0·75 (<0·001), respectively. The κ tests for the Na measurements were 0·34, which represented a fair strength of agreement, while K measurements were 0·48, which represented a moderate strength of agreement.

**Table 2.** Concordance analyses between day 1 and 2 urine collections on the sodium and potassium results* (*n* 380) (Numbers and percentages)

Measurements of urine excretion	Day 2 urine collection															
	Na excretion								K excretion							
	Low tertile		Median tertile		High tertile		Total		Low tertile		Median tertile		High tertile		Total	
	*n*	%	*n*	%	*n*	%	*n*	%	*n*	%	*n*	%	*n*	%	*n*	%
Day 1 urine collection																
Low tertile	80	21·0	41	10·8	9	2·4	130	34·2	107	28·2	30	7·9	6	1·6	143	37·6
Median tertile	38	10·0	56	14·7	31	8·2	125	32·9	23	6·1	64	16·8	31	8·2	118	31·1
High tertile	13	3·4	29	7·6	83	21·8	125	32·9	8	2·1	30	7·9	81	21·3	119	31·3
Total	131	34·5	126	33·2	123	32·4	380	100·0	138	36·3	124	32·6	118	31·1	380	100·0
Mean difference	3·1								0·8							
95 % CI	–0·96, 7·07								0·05, 1·5							
Limits of agreement	–75, 81								–13·3, 14·9							
*r*	0·6								0·8							
*P*	<0·001								<0·001							
*κ* †	0·34								0·48							

*r*, Correlation coefficient; κ, agreement κ.

* This analysis conducted only with two successful urine collections were included in the analyses.

† To measure the concordance among day 1 and 2 measurements of 24-h urine, we used Bland–Altman, linear regression, Spearman’s correlation and Cohen’s κ test (κ).

Data on the excretion of Na and K are shown in Table 3. The mean values for Na and K excretions were 102·8 and 25 mmol/d in men and 100·6 and 23·4 mmol/d in women, respectively. Na and K excretion results by age, BMI and PAL are summarised in Table 4. Mean values of Na and K excretions were higher in younger participants, those with higher BMI and those with higher PAL. The Na:K ratios in both men and women were also higher in younger participants and in those with a higher BMI. In men, the Na:K ratio was higher in those with lower PAL, whereas in women the Na:K ratio was higher in those with a higher PAL.

**Table 3.** Results of 24-h urine collection by sex (Mean values and standard deviations)

	Men (*n* 240)		Women (*n* 239)		Total (*n* 479)	
	Mean	sd	Mean	sd	Mean	sd
Collected volume (ml)	1036·4	482·6	1014·0	469·0	1025·2	475·5
Creatinine excretion (mg/d)	1003·7	343·4	727·1	222·0	865·7	320·4
Creatinine ratio (%)*	101·3	30·2	72·6	19·4	87·0	29·2
Na excretion (mmol/d)	102·8	43·7	100·6	36·4	101·5	40·3
Na excretion (mmol/g Cr)†	108·9	51·3	139·8	44·0	125·4	51·6
K excretion (mmol/d)	25·0	8·9	23·4	8·2	24·2	8·6
K excretion (mmol/g Cr)†	26·6	10·8	33·1	11·4	30·1	12·0
Na:K ratio‡	6·6	2·7	6·7	2·6	6·7	2·7

* The ratio of observed to expected creatinine excretion calculated using the equations of Joossens *et al.* If the ratio was <60 % or >140 %, the collection was considered unsuccessful.

† The unit (mmol/g Cr) indicated the excretion of the electrolyte (mmol) per 1 g excretion of creatinine.

‡ Ratio of Na (mg/d):K (mg/d).

**Table 4.** Results of 24-h urine collection by sex and other factors (age, BMI and physical activity level (PAL); *n* 479) (Mean values and standard deviations)

			BMI (kg/m^2^)		Collected volume (ml)		Creatinine excretion (mg/d)		Creatinine ratio (%)*		Na excretion (mmol/d)		K excretion (mmol/d)		Na:K ratio†	
Sex	Categories	*n*	Mean	sd	Mean	sd	Mean	sd	Mean	sd	Mean	sd	Mean	sd	Mean	sd
Men (*n* 240)	Age (years)															
	20–39	34	21·5	4·3	1068·9	481·3	1208·0	372·2	118·6	36·4	111·6	39·5	22·1	8·3	8·4	3·6
	40–59	85	21·9	3·7	1134·2	521·0	1090·2	348·3	104·2	32·1	106·8	47·6	26·3	8·7	6·4	2·7
	60–79	110	20·3	3·3	979·0	444·5	921·1	268·6	97·2	23·0	99·6	42·5	25·2	9·3	6·3	2·4
	80–99	11	17·7	2·2	753·0	399·2	529·4	167·7	67·0	22·0	71·8	22·8	19·9	7·8	5·8	1·6
	BMI (kg/m^2^)															
	Underweight (<18·5)	64	16·9	1·4	869·7	389·5	835·9	296·2	103·2	29·7	83·3	34·9	22·1	8·2	6·2	2·8
	Normal (18·5–24·9)	146	21·2	1·7	1091·1	511·7	1024·3	312·6	101·8	31·2	104·3	41·1	25·3	9·2	6·7	2·8
	Overweight (25·0–29·9)	27	27·0	1·4	1109·6	441·1	1212·4	383·0	94·4	27·5	128·0	48·8	27·9	7·3	7·0	2·4
	Obesity (> 30)	3	35·4	3·7	1267·5	536·9	1702·7	285·0	101·5	12·6	199·2	59·1	39·3	8·5	7·8	2·3
	PAL (MET × h)‡															
	Q1 (9·6)	76	21·4	3·8	993·1	507·4	949·3	356·7	93·6	28·3	97·0	38·6	23·3	9·1	6·8	2·7
	Q2 (41·6)	45	20·6	4·2	1095·9	489·0	977·4	258·6	102·5	22·1	114·5	49·8	26·0	9·4	7·0	2·9
	Q3 (80·2)	61	21·2	3·6	1058·0	497·3	1016·6	343·6	100·9	29·5	101·1	38·8	25·2	7·9	6·4	2·8
	Q4 (148·9)	58	20·2	3·1	1024·1	432·5	1081·8	374·4	111·1	36·2	102·1	49·5	26·0	9·4	6·2	2·8
Women	Age (years)															
(*n* 239)	20–39	35	24·8	4·4	1108·1	436·7	895·5	208·9	83·3	21·5	116·7	29·4	21·6	6·6	8·8	3·3
	40–59	84	24·8	4·0	1053·4	480·1	792·1	193·4	75·0	17·7	114·8	38·1	24·8	8·0	7·3	2·5
	60–79	112	22·8	4·1	961·3	467·7	639·4	200·1	67·9	18·4	86·7	30·7	23·2	8·7	5·9	1·9
	80–99	8	19·9	2·8	927·2	483·5	535·0	167·5	68·3	21·8	70·8	29·1	19·8	5·2	5·3	1·4
	BMI (kg/m^2^)															
	Underweight (<18·5)	29	16·6	1·4	855·0	470·0	545·1	199·9	76·9	26·7	76·1	26·2	18·5	5·1	6·4	2·7
	Normal (18·5–24·9)	124	22·2	1·7	1008·0	467·9	695·2	195·6	74·3	19·0	94·2	31·9	22·9	7·6	6·6	2·6
	Overweight (25·0–29·9)	72	27·6	1·4	1056·9	480·1	825·1	211·6	69·7	16·8	115·3	40·0	25·3	9·3	7·2	2·6
	Obesity (> 30)	14	31·5	1·1	1176·1	354·5	882·6	216·3	64·3	14·4	128·5	23·9	28·0	6·8	7·1	1·8
	PAL (MET × h)‡															
	Q1 (19·8)	45	23·5	4·6	1020·3	541·8	663·8	245·8	68·1	19·0	88·8	34·4	22·4	8·2	6·1	2·0
	Q2 (40·7)	74	24·3	4·1	1037·3	474·0	715·6	225·9	69·3	18·7	101·0	37·9	22·8	7·6	6·9	2·5
	Q3 (74·3)	60	23·4	4·2	1041·4	437·6	725·7	212·8	72·9	19·6	104·1	36·1	23·8	9·4	7·1	2·9
	Q4 (160·1)	60	23·3	4·2	953·2	440·1	790·1	195·1	79·9	18·8	104·6	35·1	24·5	7·5	6·7	2·6

MET, metabolic equivalents; Q, quantile.

* The ratio of observed to expected creatinine excretion calculated using the equations of Joossens *et al*. If the ratio was <60 % or >140 %, the collection was considered unsuccessful.

† Ratio of Na (mg/d):K (mg/d).

‡ PAL categorised into quartiles, and mean PAL for each category was shown in parentheses.

Table 5 shows the results of the multivariate linear regression analysis. Age was significantly associated with a lower Na excretion in women but not in men. For both sexes, the BMI was significantly associated with a higher Na excretion and the BMI and PAL were associated with higher K excretions. For habitual intake (Table 6), none of the K intakes met the recommended value in both sexes. However, 20 % of participants met the salt intake recommendation. Additionally, there were no significant differences between groups in environments of lower and higher temperatures and humidities with urine excretion (online Supplemental Material 1).

**Table 5.** Associations between age, BMI, physical activity level (PAL) and excretion of sodium and potassium by sex*

		Na			K			Na:K ratio†		
Sex	Variables	Regression coefficient	Standardised partial regression coefficient	*P*	Regression coefficient	Standardised partial regression coefficient	*P*	Regression coefficient	Standardised partial regression coefficient	*P*
Men	Age (years)	–0·26	–0·09	0·16	0·08	0·14	0·03	–0·05	–0·28	<0·001
(*n* 240)	BMI (kg/m^2^)	4·77	0·40	<0·001	0·78	0·32	<0·001	0·04	0·06	0·37
	PAL (MET × h)	0·01	0·01	0·85	0·02	0·14	0·03	–0·01	–0·15	0·02
Women	Age (years)	–0·75	–0·30	<0·001	0·06	0·12	0·08	0·06	0·12	0·08
(*n* 239)	BMI (kg/m^2^)	2·77	0·32	<0·001	0·69	0·36	<0·001	0·69	0·36	<0·001
	PAL (MET × h)	0·03	0·05	0·40	0·02	0·13	0·04	0·02	0·13	0·04

MET, metabolic equivalents.

* A multivariable linear regression model analysed the association of age, BMI and PAL on Na and K excretions. The dependent variable was Na or K excretion, and the independent variable was age, BMI and PAL, which were continuous variables and were included in the model simultaneously. The coefficient showed how much the dependent variable would typically change given a one standard deviation change in each independent variable. Standardised partial regression was also shown to compare the relative impact of each of the covariates quantitatively.

† Ratio of Na (mg/d):K (mg/d).

**Table 6.** Estimated proportion of participants whose sodium and potassium intakes met the recommended values (<5 g/d) in the population analysed* (*n* 380)

				Probability of meeting the recommended values adjusted for excretion (%)		
	Recommended by	Recommended values for nutrient intake (mmol/d)	Recommended values adjusted for excretion (mmol/d)†	Total	Men (*n* 204)	Women (*n* 176)
Na‡	Indonesia government and WHO	85·0	73·1	19·7	22·5	16·5
K§		90·0	69·3	0·0	0·0	0·0

* This analysis conducted only with two successful urine collections were included in the analyses. The estimation of habitual Na and K intakes was simulated using HabitDist software.

† These values were divided by 0·86 for Na and 0·77 for K to apply the recommendations for dietary intake to the excretion data^(61)^.

‡ 1 mmol Na = 23·0 mg Na = 58·5 mg NaCl.

§ 1 mmol K = 39·1 mg K.

## Discussion

To the best of our knowledge, this is the first study to investigate Na and K excretions using 24-h urine collection, a ‘gold standard’ method^(65)^, in Indonesians based on a representative sample in a city of Indonesia. We collected urine samples over two 24-h periods to determine the Na and K excreted. The results of the concordance analysis showed a good agreement between the two measurements (days 1 and 2) even though considerable day-to-day variations existed in both the Na and K excretions into 24-h urine. Therefore, the 24-h urine data were confirmed as reliable for use in the analysis.

In the present study, mean Na intake was 102·8 mmol/d (6 g NaCl) in men and 100·6 mmol/d (5·9 g NaCl) in women. These findings are higher than the recommended values of <73·1 mmol/d (5 g NaCl) for Na excretion^(66)^. Previous studies have reported that the Na intake among Indonesian was 6·9 g NaCl (*n* 15, adult women in urban area)^(27)^ and 6·16 g NaCl (*n* 51, older adult women in coastal area)^(28)^. Both studies calculated the Na intake using a single 24-h urine excretion. A previous study that used 24-h dietary recall reported higher values than the 24-h urine collection, that is, 9·4 g NaCl (*n* 395 women in urban area)^(26)^. This present study has similar result with the previous Indonesian which used 24-h urine excretion. Notwithstanding, the awareness of Indonesian people of the benefits of avoiding salty food is quite high, at 68·6 %; however, the average Na intake is still high^(28)^, suggesting that awareness alone is not enough to decrease salt intake. While most people know that they should reduce their salt intake, there may be confusion as to which foods should be avoided. Therefore, an exploration of commonly consumed Indonesian foods (dietary habit) with high Na content is required.

While the Indonesian Na intake is high, other East Asian countries (Korea, China and Japan) reported a higher Na intake than that observed in the present study^(18,23,67–69)^. We suggest two reasons to explain these results. First, the characteristics of foods eaten commonly in other East Asian countries and in Indonesia may have influenced the intake of Na^(23,56)^, while Indonesian people usually consume noodle products, salty seafood products, composite foods, foods with coconut milk and fried foods which contain high Na^(70)^. However, people from other East Asian countries may consume more high-Na-containing foods than Indonesians. For example, Japanese people consume miso and soya sauce, both of which are very high in Na. Second, a previous study found that 14 % of Na intake was excreted in faeces and sweat^(33)^. This percentage is expected to be higher in a tropical country such as Indonesia with a mean temperature of 27·5°C and humidity of 56·9 %. The mean temperature in other East Asian countries is lower than that in Indonesia. Thus, there is a possibility that the actual Na intake is higher than the observed Na intake in the present study.

The mean K excretion among Indonesian people was lower than the recommended values for K intake^(64)^. Previous studies in Indonesia reported that the K intake among older adults was 48·5 mmol/d (1895 mg K) in men, 41·8 mmol/d (1634 mg K) in women^(26)^, and among adult diabetic patients, it was 37·4 mmol/d (1460 mg K)^(71)^, which is higher than that reported in the present study. This may have been because those previous studies used 24-h dietary recall and semi-quantitative FFQ, which tends to overestimate. Nevertheless, that result was still below the recommended values for K intake. The national survey by Indonesia’s government showed that almost all Indonesians consumed, per d, fewer than five servings of fruits/vegetables, which are high in K^(36)^. Indonesian people would be needed to consume more food containing K.

A multivariate linear regression showed that the BMI was significantly associated with Na and K excretion in both sexes. A higher BMI indicated a higher food consumption, and higher food consumption reflects higher nutrient intake^(14,72)^. Individuals with a higher BMI tend to have unhealthier lifestyles than those with a lower BMI, including a preference for salty foods^(73)^. Careful attention should be paid to individuals with a high BMI.

Age was associated with Na excretion in both sexes with K excretion and the Na:K ratio only among men. Younger and older adults have different preferences for food selection. The younger select more variety and modern food (i.e. bread, noodle, confectioneries and meat) which is known to result in a higher Na intake^(56,74,75)^. Furthermore, a previous study showed that younger age, male sex and smoking were associated with a less healthy diet^(74,76)^. Because of the high prevalence of non-communicable diseases in older adults, some older adults may have had the opportunity to receive healthy diet education^(77)^, which could have changed their eating habits. Younger adults would have had less opportunity to receive education regarding healthy diets, and younger age may have been related to the higher Na and lower K excretion in the present study. Thus, the target population for the salt education programme should be expanded to younger adults.

In the present study, more than 80 % of the participants were estimated to consume more Na than the recommended value (<5 g/d). For K intake, no participant (0 %) met the recommended value (>3510 mg/d). Therefore, there is an urgent need to develop interventions to reduce Na intake and to increase K intake. In Indonesia, there are Integrated Guidance Posts (*Posbindu*), wherein all community-dwelling people gather at least once a month to measure their blood pressure and body weight^(78)^. Thus, conducting a salt reduction programme in the Integrated Guidance Posts may be an effective intervention for people in the community.

The present study had several limitations. First, to minimise any errors and to improve the feasibility of obtaining enough samples, we decided to collect data in one city, and all the accumulated urine was analysed at the same clinical testing company. Second, some participants had a history of other diseases and may have taken medications that had an effect on Na and K excretion. To confirm the assumption, we recalculated the Na and K excretions after excluding the participants with hypertension based on blood measurement and/or those who took antihypertensive and diuretic drugs (*n* total = 297; men = 149 and women = 148; data not shown). The mean values for Na and K urine excretions were 99·7 and 24·6 mmol/d in men, and 100·8 and 22·5 mmol/d in women, respectively. These excretion values were almost the same as those obtained in our study population. Third, the biomarkers measured in the urine sample were Cr, Na and K. However, on urine collection day 2, we only measured urine Na and K, due to budget constraints. Therefore, we used a different method to measure the successful urine collection in the 24-h urine collection between days 1 and 2. However, both methods have been used and validated in previous studies^(48,54,55)^. Fourth, the independent variables in the present study were reported as factors related to energy intake which was the major predictor of salt intake. However, the association between the variables and salt intake in each population and in the country was slightly different in the previous studies^(18,19,79)^.

In conclusion, we estimated Na and K intakes among community-dwelling adults in an Indonesian city and found a significantly positive association of BMI with Na excretion. The mean salt intake among the participants was estimated to be higher than the values recommended by the Indonesian government. Further, the K intake was lower compared with the recommended values. From the estimation of habitual Na and K intakes, the number of participants who met the recommended value was low. In the future, it will be essential to consider the Indonesian food dietary patterns when intervention programmes against high Na and low K are planned in this country.

## Supplementary material

For supplementary material accompanying this paper visit https://doi.org/10.1017/S0007114521000271. click here to view supplementary material
