# Supplementary material for: Estimation of sodium and potassium intakes assessed by two 24-hour urine collections in a city of Indonesia
Source: Br J Nutr. 2021 Jan 26;126(10):1537–48. doi: 10.1017/S0007114521000271 (PMC8524422; doi:10.1017/S0007114521000271)
Supplement: Supplementary file 1 [file S0007114521000271sup001.docx]

| **Supplemental Material 1. The effect of environment condition with the result of urine in tropic country** | | | | | | | | | | | | | | | |  |  |  |  |  |  |  |
| --- | --- | --- | --- | --- | --- | --- | --- | --- | --- | --- | --- | --- | --- | --- | --- | --- | --- | --- | --- | --- | --- | --- |
| (Mean values and standard deviations, n=479) | | | | | | |  |  |  |  |  |  |  |  |  |  |  |  |  |  |  |  |
|  |  |  | **BMI** | |  | **Collected volume** | |  | **Creatinine**  **excretion** | |  | **Creatinine ratio** | |  | **Na excretion** | |  | **K excretion** | |  | **Na:K ratio^†^** | |
|  |  |  | **(kg/m^2^)** | |  | **(ml)** | |  | **(mg/d)** | |  | **(%) *** | |  | **(mmol/d)** | |  | **(mmol/d)** | |  |  |  |
| **Sex** | Categories | n | Mean | SD |  | Mean | SD |  | Mean | SD |  | Mean | SD |  | Mean | SD |  | Mean | SD |  | Mean | SD |
| **Men** | Temperature^‡^ |  |  |  |  |  |  |  |  |  |  |  |  |  |  |  |  |  |  |  |  |  |
| **n= 240** | < 27,4 ºC | 121 | 20.7 | 2.9 |  | 1080.3 | 502.1 |  | 992.6 | 320.1 |  | 101.6 | 29.8 |  | 103.5 | 43.2 |  | 25.2 | 8.6 |  | 6.6 | 2.8 |
|  | ≥ 27,5 ºC | 119 | 21.1 | 4.3 |  | 991.6 | 459.8 |  | 1015.0 | 366.6 |  | 101.1 | 30.8 |  | 101.6 | 44.7 |  | 24.6 | 9.4 |  | 6.6 | 2.7 |
|  | *P value* |  | 0.34 | |  | 0.16 | |  | 0.61 | |  | 0.92 | |  | 0.74 | |  | 0.64 | |  | 0.88 | |
|  | Humidity^‡^ |  |  |  |  |  |  |  |  |  |  |  |  |  |  |  |  |  |  |  |  |  |
|  | < 56.4% | 108 | 21.0 | 4.1 |  | 1006.7 | 486.5 |  | 979.0 | 380.0 |  | 98.9 | 31.8 |  | 106.3 | 44.4 |  | 24.8 | 8.3 |  | 6.8 | 2.9 |
|  | ≥ 56.5% | 132 | 20.8 | 3.3 |  | 1060.6 | 480.0 |  | 1023.9 | 310.3 |  | 103.4 | 28.9 |  | 99.5 | 43.3 |  | 25.0 | 9.6 |  | 6.4 | 2.7 |
|  | *P value* |  | 0.66 | |  | 0.39 | |  | 0.31 | |  | 0.25 | |  | 0.23 | |  | 0.90 | |  | 0.30 | |
| **Women** | Temperature^‡^ |  |  |  |  |  |  |  |  |  |  |  |  |  |  |  |  |  |  |  |  |  |
| **n= 239** | < 27,4 ºC | 134 | 23.2 | 4.2 |  | 1004.1 | 448.5 |  | 705.4 | 235.6 |  | 71.9 | 20.5 |  | 100.1 | 33.3 |  | 23.5 | 8.7 |  | 6.8 | 2.5 |
|  | ≥ 27,5 ºC | 105 | 24.3 | 4.2 |  | 1026.6 | 495.8 |  | 754.8 | 201.0 |  | 73.5 | 18.0 |  | 100.7 | 40.1 |  | 23.3 | 7.5 |  | 6.7 | 2.6 |
|  | *P value* |  | 0.05 | |  | 0.71 | |  | 0.09 | |  | 0.53 | |  | 0.90 | |  | 0.83 | |  | 0.72 | |
|  | Humidity^‡^ |  |  |  |  |  |  |  |  |  |  |  |  |  |  |  |  |  |  |  |  |  |
|  | < 56.4% | 74 | 23.6 | 4.3 |  | 1038.6 | 521.8 |  | 685.2 | 196.9 |  | 69.5 | 17.5 |  | 96.6 | 34.1 |  | 23.5 | 8.4 |  | 6.4 | 2.1 |
|  | ≥ 56.5% | 165 | 23.7 | 4.2 |  | 1003.0 | 444.6 |  | 745.9 | 230.5 |  | 74.0 | 20.1 |  | 102.1 | 37.3 |  | 23.4 | 8.1 |  | 6.9 | 2.7 |
|  | *P value* |  | 0.80 | |  | 0.59 | |  | 0.05 | |  | 0.09 | |  | 0.28 | |  | 0.91 | |  | 0.14 | |
| The significant different was measured using Student’s t-test analysis | | | | | | | | | | | | | | | | | | | | | | |
| * The ratio of observed to expected creatinine excretion calculated using the equations of Joossens et al., If the ratio was < 60% or > 140%, the collection was considered unsuccessful. | | | | | | | | | | | | | | | | | | | | | | |
| ^†^ Ratio of Na (mg/d) : K (mg/d) | | |  |  |  |  |  |  |  |  |  |  |  |  |  |  |  |  |  |  |  |  |
| ^‡^ Grouping variable based on the median of each variable | | | | | | |  |  |  |  |  |  |  |  |  |  |  |  |  |  |  |  |
